# Artificial Intelligence for Radiographic Diagnosis of Peri-Implantitis: A Comprehensive Review on Detection, Measurement, and Risk Stratification

**DOI:** 10.3390/jcm15135210

**Published:** 2026-07-03

**Authors:** Francesco Fanelli, Angela Tisci, Lorenzo Lo Muzio, Giuseppe Troiano, Vito Carlo Alberto Caponio, Mario Dioguardi, Khrystyna Zhurakivska

**Affiliations:** 1 Department of Clinical and Experimental Medicine, University of Foggia, 71122 Foggia, Italy; angela.tisci@unifg.it (A.T.); lorenzo.lomuzio@unifg.it (L.L.M.); mario.dioguardi@unifg.it (M.D.); khrystyna.zhurakivska@unifg.it (K.Z.); 2 Department of Medicine and Surgery, LUM University, 70010 Casamassima, Italy; troiano@lum.it; 3 Department of Life Sciences, Health and Healthcare Professions, Link Campus University, 00165 Rome, Italy; vca.caponio@unilink.it

**Keywords:** peri-implantitis, artificial intelligence, deep learning, radiography, dental, alveolar bone loss

## Abstract

**Background/Objectives**: Peri-implantitis is a major complication in implant dentistry, and its radiographic diagnosis remains challenging because conventional assessment is operator-dependent and bone loss is often detected only after measurable changes occur. Artificial intelligence (AI) may support the detection, quantification, and prognostic assessment of peri-implant bone conditions. This review aimed to synthesize evidence on AI-based radiographic approaches for peri-implantitis detection, marginal bone loss measurement, and risk stratification. **Methods**: PubMed and Scopus were searched for original studies published between 2013 and 2025 that applied artificial intelligence (AI), including machine learning and deep learning, to peri-implantitis. Eligible studies focused on peri-implant bone assessment and reported quantitative performance metrics. Extracted data included imaging modality, AI model, task, dataset, reference standard, validation strategy, performance, and clinical relevance. A qualitative synthesis was performed. **Results**: Eleven studies met the eligibility criteria; however, one full text could not be retrieved, and ten studies were included. In most of the studies, peri-implant marginal bone loss detection or measurement was performed using periapical/intraoral radiographs, while only few studies used panoramic or combined imaging. Common architectures included YOLO variants, Faster R-CNN, Mask R-CNN, U-Net, ResNet, and AlexNet. Performance was generally encouraging for implant localization, bone loss detection, keypoint identification, and severity classification. Only one study addressed outcome prediction. All studies were retrospective and internally validated. **Conclusions**: AI may support radiographic detection and quantification of peri-implant bone loss as an adjunctive diagnostic tool. However, evidence is limited by retrospective designs, heterogeneous reference standards, lack of external validation, and limited clinical-data integration. Future studies should prioritize prospective multicenter validation, longitudinal imaging, and multimodal models.

## 1. Introduction

Peri-implantitis represents one of the most significant complications in contemporary implant dentistry, with recent meta-analyses indicating that approximately one in five patients with dental implants develops this condition [1]. The clinical burden extends beyond prevalence statistics, as peri-implantitis constitutes the primary reason for implant explantation in 62% of cases [2], underscoring an urgent need for enhanced diagnostic strategies capable of early disease identification and intervention.

Current diagnostic approaches rely predominantly on clinical parameters, including probing depth measurements and bleeding on probing, supplemented by radiographic assessment. However, these conventional methods demonstrate substantial limitations. Bleeding on probing exhibits a positive predictive value of merely 24.1% for peri-implantitis diagnosis [3], while the absence of a single definitive diagnostic tool necessitates reliance on parameter combinations that often fail to distinguish between stable and progressive disease states [4]. Standard intraoral radiography, though remaining the gold standard for monitoring marginal bone levels, requires meticulous standardization and can only detect changes once measurable bone loss has occurred [5]. These inherent constraints create a compelling rationale for developing objective, reproducible diagnostic approaches [6].

Artificial intelligence (AI), particularly deep learning, has emerged as promising innovations to address these diagnostic limitations. Systematic evaluations demonstrate that convolutional neural network (CNN) architectures can achieve diagnostic accuracies ranging from 73% to 99% for peri-implant bone loss detection [7], with pilot investigations employing faster region-based CNN architecture reporting moderate to substantial agreement with expert assessments [8]. These AI-based systems offer the fundamental advantage of providing consistent, operator-independent assessments that may enhance diagnostic standardization and reduce inter-observer variability in radiographic interpretation.

Beyond binary disease detection, the clinical utility of AI extends to quantitative measurement and severity assessment of peri-implant bone loss. Accurate quantification remains essential for staging, treatment planning, and longitudinal monitoring, yet conventional manual measurement suffers from subjectivity and limited reproducibility. Advanced imaging modalities, including cone beam computed tomography, offer three-dimensional visualization capabilities that, when coupled with AI algorithms, demonstrate diagnostic accuracy approaching 80%, with area under the receiver operating characteristic curve values of 88% [9]. Furthermore, the integration of multiple risk factors into machine learning-based prognostic models has shown promise for individualized risk stratification, with deep learning approaches consistently achieving area under the curve values exceeding 0.8 for peri-implantitis prediction [10].

The translation of AI from experimental prototypes to clinical implementation requires careful consideration of current methodological limitations and validation standards. Most existing models derive from single-center, retrospective datasets with limited external validation, while substantial heterogeneity exists in outcome definitions, imaging protocols, and reported performance metrics [11]. Additionally, the fundamental constraint that radiographic bone loss must be present for algorithmic detection limits the capacity for truly early diagnosis of subclinical disease. Despite the growing number of studies investigating AI in peri-implant imaging, the available evidence remains fragmented, with substantial heterogeneity in imaging modalities, outcome definitions, algorithmic architectures, and validation methodologies. To date, no comprehensive review has systematically synthesized the evidence regarding AI-based radiographic detection, quantitative assessment, and risk stratification of peri-implantitis.

The present comprehensive review critically evaluates the current state of AI applications for radiographic diagnosis of peri-implantitis, with specific emphasis on detection methodologies, quantification approaches, and risk stratification capabilities. By systematically examining the performance characteristics, clinical applicability, and methodological limitations of existing AI systems, this review aims to provide evidence-based insights to guide future development, validation, and clinical implementation of next-generation diagnostic tools in implant dentistry.

## 2. Materials and Methods

This comprehensive review was conducted using a structured literature search, predefined eligibility criteria, independent study screening, and standardized data extraction procedures to ensure transparency and reproducibility. The objective of the review was to identify and synthesize the available evidence on the application of AI to radiographic imaging for the assessment of peri-implant bone conditions. The methodology was designed to provide a broad and critical overview of the available evidence while maintaining a structured and reproducible approach to study identification, selection, and data extraction. The review protocol was defined a priori but was not formally registered.

A comprehensive literature search was performed using two electronic databases, PubMed and Scopus, to identify relevant studies published between 1 January 2013 and 31 December 2025. The search strategy was developed using combinations of keywords related to dental implants, peri-implant conditions, AI, and radiographic imaging modalities. The full search strategies for each database are reported in Table 1. The search was conducted on 20 March 2026. In addition, reference lists of selected review articles were manually screened to identify potentially relevant studies through backward citation tracking.

Eligibility criteria were defined a priori and are summarized in Table 2. Briefly, studies were included if they investigated the application of AI to radiographic imaging of dental implants with a focus on peri-implant bone conditions, and if they reported quantitative performance metrics. Studies not meeting these criteria or lacking sufficient methodological detail were excluded.

All retrieved records were imported into reference management software (Bookends version 14.2 Sonny Software, Virginia Beach, VA, USA), and duplicate entries were identified and removed. The remaining records were screened based on title and abstract by two independent reviewers. Full-text articles were subsequently assessed for eligibility by the same reviewers. Any disagreements during the screening or eligibility assessment process were resolved through discussion and consensus. No automation tools were used during the selection process.

Data extraction was performed independently by two reviewers using a predefined and standardized data extraction form. The extracted data included bibliographic information (author and year of publication), imaging modality, type of task (detection, segmentation/measurement, or prediction), study objective, AI model, dataset characteristics (including sample size), reference standard, study design, validation strategy, and reported performance metrics and results. Additional variables, such as clinical relevance and the use of explainability techniques, were also collected when available. In cases of missing or unclear information, no assumptions were made and the data were reported as not specified.

Given the heterogeneity in study design, imaging modalities, datasets, and outcome measures, a quantitative meta-analysis was not performed. Instead, a qualitative synthesis was conducted. Studies were grouped according to the type of task addressed (detection, segmentation/measurement, or prediction), and results were summarized using descriptive tables and narrative analysis. Differences across studies were explored qualitatively by comparing methodological aspects, including model architecture, dataset characteristics, and validation strategies. No formal sensitivity analyses were conducted.

To provide a structured methodological quality appraisal of the included AI studies, a CLAIM-based assessment was performed using the 42-item Checklist for Artificial Intelligence in Medical Imaging. Included studies were evaluated item by item, and each criterion was scored as reported (1), not reported (0), or not applicable (N/A). CLAIM compliance was calculated for each study as the number of reported items divided by the number of applicable items, with non-applicable items excluded from the denominator. The complete item-level CLAIM assessment is reported in Supplementary Table S1.

CLAIM appraisal was used to support the interpretation of the reliability, transparency, reproducibility, and clinical applicability of the reported AI performance metrics. No formal certainty-of-evidence assessment was performed because of the limited number of the included studies and the substantial methodological heterogeneity of the available evidence.

In addition to database searching, backward citation tracking was performed using selected review articles.

## 3. Results

### 3.1. Study Selection

**Figure 1:**
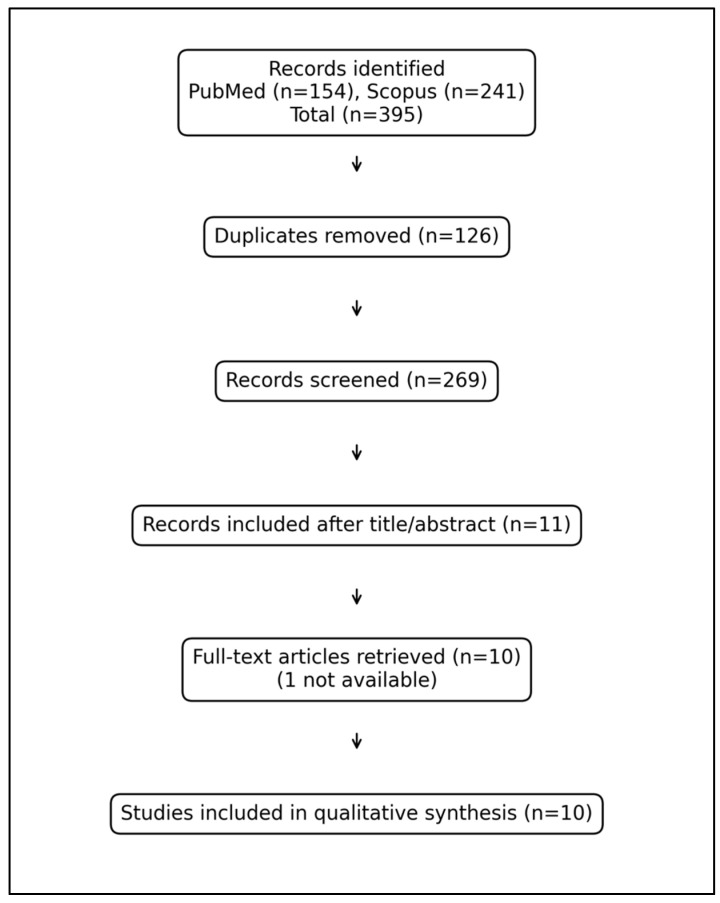
Flow diagram of the study selection process. A total of 395 records were identified through database searching (PubMed, n = 154; Scopus, n = 241). After removal of 126 duplicates, 269 records were screened based on title and abstract. Eleven studies met the eligibility criteria and were selected for full-text assessment. Of these, 10 full-text articles were successfully retrieved, while 1 study could not be accessed despite attempts to contact the authors. Ultimately, 10 studies were included in the qualitative synthesis.

A total of 395 records were identified through database searching, including 154 records from PubMed and 241 from Scopus. All retrieved records were exported into a reference management software (Bookends), and duplicate entries (n = 126) were identified and removed. After duplicate removal, 269 records were screened based on title and abstract. Among the 269 screened records, 48 review articles were excluded from eligibility assessment as non-original studies but were retained for background synthesis and backward citation tracking. Study selection was conducted in two phases: initial screening of titles and abstracts, followed by full-text assessment of potentially relevant articles. In addition to database searching, backward citation tracking was performed using selected review articles. Thirteen review articles were selected for backward citation tracking. This process identified one additional potentially relevant study. However, this study was excluded after full-text evaluation because it did not report quantitative performance outcomes relevant to peri-implant bone assessment. Following the selection process, 11 studies met the eligibility criteria and were selected for full-text assessment. Of these, 10 full-text articles were successfully retrieved, while one study could not be accessed despite attempts to contact the corresponding authors and was therefore excluded. Ultimately, 10 studies were included in the final qualitative synthesis. The study selection process is summarized in Figure 1.

### 3.2. Study Characteristics

The main characteristics of the included studies (n = 10) are summarized in Table 3. All studies were published between 2021 and 2025 and investigated the application of artificial intelligence to the radiographic assessment of peri-implant bone conditions. Most studies were based on periapical or intraoral radiographs, including those by Cha et al., Chen et al., Gao et al., Lee et al. (2024), Liu et al., Mao et al., and Vera et al., [8,12,13,14,15,16,17] whereas fewer studies used panoramic radiographs or a combination of periapical and panoramic imaging modalities, as reported by Kibcak et al., Lee et al. (2025), and Zhang et al. [18,19,20].

From a methodological perspective, most studies addressed detection and measurement tasks, frequently combined with severity classification of peri-implant bone loss. These approaches aimed to automatically localize implants, peri-implant bone defects, implant landmarks, or anatomical keypoints, and to quantify marginal bone loss through bounding-box detection, keypoint localization, segmentation, or hybrid image-processing pipelines. Cha et al. used a modified Mask R-CNN framework to identify implant-related keypoints and automatically calculate the ratio between peri-implant bone loss and implant length. Gao et al. applied YOLOv8-pose for automated keypoint detection and marginal bone loss measurement on periapical radiographs. Similarly, Vera et al. combined YOLOv3-based implant localization with geometric image-understanding procedures to quantify peri-implant marginal bone remodeling, whereas Mao et al. integrated YOLOv8 with image-processing algorithms to classify disease severity according to exposed implant threads.

Object-detection architectures were the most frequently adopted models, particularly YOLO variants and Faster R-CNN. Segmentation and classification networks, including U-Net, Mask R-CNN, ResNet, and AlexNet, were also used across the included studies. Chen et al., Mao et al., and Vera et al. adopted hybrid approaches combining deep learning with rule-based or image-processing methods to improve measurement accuracy, workflow automation, or clinical interpretability. In contrast, Zhang et al. was the only study focused on outcome prediction rather than radiographic detection or measurement, using a ResNet-50-based hybrid model to classify implant success and failure from periapical and panoramic radiographs.

Dataset size varied substantially across studies, ranging from a few hundred radiographs to several thousand images, with considerable heterogeneity in imaging modality, annotation strategy, data composition, and reporting of patient-level information. Ground-truth annotations were generally provided by experienced clinicians, often through manual labeling of implants, bone levels, peri-implant defects, or regions of interest, sometimes using consensus procedures or multiple expert evaluators. All included studies had a retrospective design, and validation strategies were consistently limited to internal validation, including train–validation–test splits or cross-validation; no study performed external validation on an independent dataset.

Performance metrics were heterogeneous and included accuracy, sensitivity, specificity, precision, recall, F1-score, AUC, mean average precision, Intersection over Union, Dice similarity coefficient, and keypoint localization error. Overall, most studies reported encouraging diagnostic or measurement performance, particularly for automated detection, segmentation, and severity classification of peri-implant bone loss. In some cases, AI-based systems showed performance comparable to or exceeding that of clinicians, especially for standardized detection and classification tasks, as reported by Cha et al., Liu et al., Mao et al., and Lee et al. (2025) [8,12,13,20]. However, despite these promising results, several methodological limitations were consistently observed across studies, including retrospective design, lack of external validation, relatively small or single-center datasets in several investigations, heterogeneous reference standards, and exclusive reliance on radiographic data without systematic integration of clinical parameters. Therefore, the frequently high reported performance should be interpreted with caution, as the predominance of internally validated retrospective models limits the generalizability and immediate clinical applicability of the proposed AI systems.

### 3.3. CLAIM-Based Quality Assessment (Checklist for Artificial Intelligence in Medical Imaging)

The CLAIM-based methodological quality appraisal is summarized in Table 4. CLAIM compliance ranged from 67.5% to 82.5%, with a mean compliance of 73.3%, a median of 72.8%, and a standard deviation of 4.4%. Vera et al. showed the highest compliance score (33/40; 82.5%), followed by Gao et al. (31/40; 77.5%). The lowest compliance was observed for Liu et al. (27/40; 67.5%).

Overall, core methodological and reporting items, including study objectives, data sources, model architecture, training details, model selection, performance metrics, study limitations, clinical implications, and funding/conflict-of-interest statements, were consistently reported. Conversely, several items related to transparency, reproducibility, and generalizability were poorly reported. No study performed external validation, reported demographic and clinical characteristics by data partition, provided study registration, or made a study protocol accessible. In addition, sample size justification and explainability methods were reported in only one study each, while robustness analyses and uncertainty estimates were infrequently reported. The complete item-level CLAIM assessment is provided in Supplementary Table S1, and item-level reporting frequencies are shown in Supplementary Figure S1.

### 3.4. Methodological Limitations of Included Studies

The included studies presented several methodological limitations, mainly related to their retrospective design, the use of single-center datasets, and the absence of external validation, as all models were evaluated using internal data splits only. Additional limitations were associated with the reference standard, which was generally based on expert annotations with limited reporting of inter-observer variability. Furthermore, all studies relied exclusively on radiographic data without integration of clinical parameters, potentially reducing the representativeness of peri-implantitis diagnosis. Some study-specific limitations were also identified. For example, Chen et al. showed a strong dependence on image preprocessing, Liu et al. reported lower performance compared with expert clinicians, and Gao et al. included a multicenter dataset but with a limited test set. Similarly, Mao et al. and Vera et al. used hybrid pipelines with improved interpretability but without external validation. Overall, the lack of external validation and limited generalizability represented the most consistent methodological limitations across the included studies.

## 4. Discussion

Artificial intelligence is emerging as a promising tool for the radiographic assessment of peri-implant conditions, particularly for identifying marginal bone loss, classifying defects, and supporting implant prognosis. The available evidence shows encouraging diagnostic performance, suggesting that these systems may improve the standardization of radiographic follow-up and reduce inter-operator interpretive variability [21]. In the more specific context of peri-implant marginal bone loss, available meta-analyses report high values of sensitivity, specificity, and AUC, indicating that AI models may represent a valuable support tool for the radiographic diagnosis of bone loss around implants. However, these tools should be interpreted as decision-support aids rather than substitutes for clinical judgment [22]. Against this background, the findings of the present review should be interpreted critically. The studies included in this review show considerable methodological heterogeneity, both in terms of AI architectures and with regard to the specific aims of the developed models. Some studies focus primarily on the detection of marginal bone loss, whereas others propose more advanced approaches based on landmark localization, keypoint detection, or automated quantification of the bone defect. In this context, Liu et al. [8] applied a Faster R-CNN model to the automatic detection of marginal bone loss on periapical radiographs, demonstrating the feasibility of object detection in the radiographic diagnosis of peri-implant conditions. Methodologically more complex approaches have been reported by Cha et al. [12], who proposed a modified Mask R-CNN framework to identify implant-related keypoints and automatically calculate the ratio between peri-implant bone loss and implant length. This strategy moves beyond simple lesion detection, bringing AI-based assessment closer to a quantitative framework that more closely reflects clinical reasoning. Furthermore, the most recent studies seem to be leaning toward even more sophisticated tasks. Gao et al. [17] utilized YOLOv8-pose for keypoint detection and the automated measurement of peri-implant marginal bone loss, while Mao et al. [13] combined YOLOv8 with traditional image processing to classify peri-implantitis severity based on the exposure of implant threads. The need for more interpretable models is instead highlighted by the study by Vera et al. [14], in which YOLOv3 was used for the preliminary localization of the implant and crown, while bone loss quantification was entrusted to geometric image-understanding procedures. This separation between automated detection and geometric measurement makes the process more transparent and potentially more acceptable for clinical use. These findings are consistent with the methodological literature on the integration of dental radiography, image analysis, and computer vision. Techniques such as preprocessing, segmentation, edge detection, intensity normalization, and automated identification of bone levels can transform radiography from a predominantly qualitative tool into a quantitative support for diagnosis, staging, and monitoring [23]. From a technical perspective, the most frequently used architectures include CNNs, YOLO, U-Net, Faster R-CNN, and Mask R-CNN, applied predominantly to periapical and panoramic radiographs. These models enable not only the classification of the presence or absence of disease, but also the localization of regions of interest and, in some cases, the quantification of bone defects [24]. From the perspective of imaging modalities, most of the included studies used periapical radiographs, confirming the central role of this modality in implant follow-up. Periapical radiographs allow better visualization of marginal bone levels than panoramic radiographs; however, they remain two-dimensional images and do not allow a complete characterization of the three-dimensional morphology of the defect [25]. Studies based on panoramic radiographs, such as those by Kibcak et al. [18] and Lee et al. 2025 [20], have the merit of exploring a more widely available imaging modality that may be useful for screening purposes. However, the use of panoramic radiographs introduces potential limitations related to distortions, anatomical superimpositions, and lower detail in the visualization of marginal bone levels, aspects that should be considered when interpreting the reported performance [26]. The direct comparison between AI and clinicians, included in the study by Lee et al. 2025 [20], represents a particularly interesting aspect. The YOLOv8-based model showed overall superior performance compared with periodontal surgeons in classifying the morphology and severity of peri-implant defects, although clinicians performed better in the most severe cases. This finding suggests that AI may be particularly useful in mild or moderate cases, in which radiographic interpretation is more uncertain and inter-operator variability may be greater. In advanced cases, however, expert clinical judgment may retain an advantage, especially when radiographic assessment must be integrated with clinical signs, implant history, and follow-up trends. Lee et al. 2024 [15] also confirm that modern object-detection models can be used not only to recognize the presence of peri-implantitis, but also to classify the severity of bone loss according to the percentage of implant-length involvement. This aspect is relevant because it brings AI closer to real-world decision-making, in which defect quantification influences diagnosis, prognosis, and treatment. A distinct contribution is provided by Zhang et al. [19], who departed from purely diagnostic models by proposing a predictive approach to implant outcomes based on the integration of periapical and panoramic radiographs. Although retrospective in design, this study introduces a prognostic perspective, aiming to identify radiographic patterns associated with implant success or failure, while distinguishing between failures with and without marginal bone loss. In a related context, although not included in the present review because it was not directly focused on peri-implantitis, the study by Troiano et al. explored the use of radiomic features extracted from intraoral radiographs to predict early peri-implant bone remodeling. This work suggests that radiographic imaging may contain quantitative information that is not immediately appreciable through conventional observation, potentially useful for identifying greater site-specific susceptibility to physiological bone remodeling and, more broadly, for developing personalized risk models [27]. This approach is consistent with broader reviews on implant prognosis, in which AI is proposed as a supportive tool for the early identification of at-risk implants through the integrated analysis of radiographic patterns and clinical variables. However, these reviews are not specifically focused on the prognosis of peri-implantitis, but rather include more heterogeneous outcomes, such as implant success or failure, implant stability, osseointegration, marginal bone loss, and implant longevity [21]. In this context, the findings of the present review highlight that truly prognostic models for peri-implantitis are still very limited, whereas most available studies remain focused on the detection and radiographic measurement of bone loss. A further critical issue concerns the size and nature of the datasets. Some studies, such as Kibcak et al. [18], used very large samples, whereas others, such as Gao et al. [17], relied on smaller but methodologically sophisticated datasets. This heterogeneity makes it difficult to directly compare performance across studies and suggests that very high values of accuracy, Dice coefficient, or AUC should be interpreted in light of dataset composition, imaging modality, and validation strategy. Dependence on image quality emerges as a cross-cutting critical issue. Several studies used preprocessing, data augmentation, or radiographic enhancement to improve the visibility of implant structures and bone margins; however, this raises the issue of model robustness when applied to suboptimal images characterized by artifacts, low resolution, or anatomical superimpositions [28]. This aspect is evident in the study by Chen et al. [16], in which preprocessing plays a central role in guiding the network toward the relevant regions. Although this strategy may improve performance, it also introduces a significant dependence on the processing pipeline and may reduce the transferability of the model to images acquired using different protocols. A further element of comparison comes from the literature on marginal bone loss around fixed prostheses, where AI has been proposed to reduce variability in manual assessment and improve the standardization of radiographic diagnosis. Although this field does not directly overlap with peri-implantitis, it confirms that automated quantification of bone loss represents a cross-cutting issue in restorative, periodontal, and implant dentistry [29]. Overall, the included studies confirm that AI can support the radiographic diagnosis of peri-implantitis, particularly through automated localization, measurement of marginal bone loss, and severity classification. However, the available evidence still indicates a substantial gap between experimental performance and real-world clinical implementation, mainly due to the lack of external validation, the heterogeneity of reference standards, and the absence of systematic integration with clinical parameters [11]. A particularly relevant aspect concerns the ability of AI models to recognize subtle radiographic patterns that may be difficult for the human eye to perceive in the early stages. This may be especially useful in implant follow-up, where small changes in marginal bone level or early signs of altered osseointegration may have important prognostic implications [24]. However, the concept of early diagnosis should be discussed with caution. Many current models are able to identify bone loss that is already radiographically visible, but not necessarily a preclinical biological stage of peri-implantitis. Therefore, a true future advancement should involve the longitudinal comparison of serial radiographs, in order to identify disease progression over time rather than being limited to the assessment of a single image [30]. Moreover, peri-implantitis cannot be reduced solely to the radiographic evidence of bone loss. It is a complex inflammatory disease in which bleeding on probing, probing depth, suppuration, soft-tissue conditions, implant history, and systemic risk factors must also be considered [25]. This limitation is consistent with what is more generally observed in periodontology, where diagnosis requires the integration of radiographic and clinical parameters. Image-only models may indeed overlook key information such as CAL, PPD, mobility, furcation involvement, smoking, diabetes, and other behavioral or systemic risk factors [31]. For this reason, one of the most relevant perspectives is represented by the development of multimodal models capable of integrating radiographs, clinical data, medical history, microbiology, salivary biomarkers, and immunological parameters. Such an approach would be more consistent with the multifactorial nature of periodontal and peri-implant diseases and could promote a true precision implant dentistry [32]. Emerging digital technologies further support this direction, showing that AI should not be limited to radiographic analysis alone. Two-dimensional radiographs, three-dimensional imaging, intraoral photographs, oral biomarkers, and electronic health records represent complementary sources of information that could potentially be integrated into more robust diagnostic and prognostic models [33]. In parallel, AI is also finding applications in other areas of digital implant dentistry, such as the automatic identification of implant systems, CBCT-based surgical planning, prosthetic design, and assessment of the risk of implant success or failure. This suggests that its future role may extend from diagnostic imaging to the entire implant workflow [34]. Despite these potential benefits, the current evidence remains limited by important methodological issues. Many studies are retrospective, single-center investigations based on small or selected datasets, with predominantly internal validation and limited representativeness of real-world daily clinical variability [11]. The CLAIM-based quality appraisal provided additional insights into the methodological maturity and reporting quality of the available literature. Although the overall reporting compliance was moderate-to-high (mean 73.3%), substantial deficiencies were identified in several domains that are particularly relevant for the clinical translation of artificial intelligence systems. Most studies adequately described their objectives, datasets, model architectures, training procedures, and performance metrics. However, important limitations emerged regarding reproducibility, transparency, and generalizability. These findings are consistent with broader concerns raised in the medical artificial intelligence literature, where methodological rigor and transparent reporting have been identified as prerequisites for reliable clinical implementation [35,36].

The most critical finding was the complete absence of external validation across all included studies. External validation is widely recognized as a fundamental step for assessing the generalizability of AI models beyond the population and environment in which they were originally developed [37]. As a result, the reported performance metrics were exclusively derived from internally validated retrospective datasets, limiting confidence in the ability of these models to generalize to different clinical settings, patient populations, imaging devices, and acquisition protocols. Consequently, exceptionally high values of accuracy, AUC, Dice coefficient, or mean average precision (mAP) should be interpreted with caution. While these metrics demonstrate strong technical performance under the specific conditions in which the models were developed and tested, they do not necessarily translate into equivalent clinical utility. Performance estimates derived from internally validated retrospective datasets may be inflated by dataset-specific characteristics, limited population diversity, spectrum bias, or unrecognized sources of information leakage. Therefore, high technical accuracy should not be interpreted as evidence of clinical effectiveness until models are prospectively evaluated and externally validated across diverse real-world settings. Similarly, none of the studies reported protocol registration or public access to study protocols, making independent verification and replication difficult.

Additional methodological concerns involved the limited reporting of uncertainty estimates, robustness analyses, and explainability techniques. Only a minority of studies evaluated model uncertainty or explored performance under varying imaging conditions, while explainability methods were almost completely absent. These findings are particularly relevant in healthcare applications, where understanding model behavior, decision pathways, and confidence estimates may be as important as achieving high predictive performance [38]. Moreover, although several studies reported excellent diagnostic or measurement performance, the absence of uncertainty estimates and robustness testing limits the ability to determine whether these results would remain stable across heterogeneous real-world radiographs.

Furthermore, only a small proportion of studies reported sample size justification, inter- or intra-rater agreement, or explicit strategies to prevent data leakage between training and testing datasets. These omissions may contribute to overly optimistic performance estimates and complicate the interpretation of reported results. Data leakage is a well-recognized source of bias in machine learning studies and may artificially inflate performance metrics when information from the training dataset inadvertently influences model evaluation [39].

Collectively, these findings suggest that the current literature is characterized by rapid technological development but remains at an early stage of methodological maturity. Future investigations should place greater emphasis on external validation, reproducibility, transparency, explainability, uncertainty reporting, and adherence to reporting frameworks such as CLAIM [35]. For future prospective clinical studies evaluating AI-assisted diagnostic workflows, reporting standards such as CONSORT-AI and SPIRIT-AI may also facilitate greater methodological rigor and reproducibility [36,40]. These steps will be essential to move from promising technical performance toward reliable and clinically meaningful implementation.

A further issue concerns the quality of the images used to train the models. In several studies, radiographs with artifacts, poor quality, multiple implants, distortions, or complex anatomical conditions are excluded; this may lead to an overestimation of performance and reduce the generalizability of the models in real-world clinical scenarios [41]. Finally, although two-dimensional radiographs are cost-effective, readily available, and routinely used in implant follow-up, they may not fully capture the complex three-dimensional morphology of peri-implant defects. In contrast, CBCT imaging has the potential to provide a more comprehensive assessment of defect configuration and extent in selected clinical situations. Nevertheless, its routine application remains constrained by higher radiation exposure, additional costs, and specific clinical indications [42]. In addition, the interpretation of the currently available evidence should take into account certain limitations of the present review. The literature search was restricted to PubMed and Scopus and did not include other databases such as Embase or Web of Science, nor dedicated searches of grey-literature sources. Although backward citation tracking was performed to enhance study identification, potentially relevant publications may not have been captured. Furthermore, the marked heterogeneity of study designs, imaging modalities, AI architectures, reference standards, and outcome measures limited direct comparisons across studies and precluded quantitative synthesis. Despite these limitations, the available evidence consistently suggests that artificial intelligence has considerable potential to improve the radiographic assessment of peri-implant conditions, particularly through the automated detection, quantification, and monitoring of peri-implant bone changes. Overall, artificial intelligence represents a promising technology to support the diagnosis, quantification, and monitoring of peri-implant conditions. However, before real clinical implementation, prospective multicenter studies, external validations, more representative datasets, shared diagnostic criteria, and greater integration between imaging, clinical parameters, and biological data will be necessary.

## Figures and Tables

**Table 1:** Database search strategies.

Database	Search Strategy	Filters/Limits
PubMed	( (“dental implant” OR “peri-implant” OR periimplant OR peri-implantitis OR “implant bone” OR “implant bone loss” OR “marginal bone loss”) AND (“artificial intelligence” OR “machine learning” OR “deep learning” OR “neural network” OR “convolutional neural network” OR CNN) AND (radiograph * OR “dental radiograph *” OR panoramic OR periapical OR orthopantomography * OR CBCT OR “cone beam”) ) AND (“1 January 2013”[Date-Publication]: “31 December 2025”[Date-Publication])	Publication date: 1 January 2013–31 December 2025
Scopus	TITLE-ABS-KEY ((“dental implant” OR “peri-implant” OR periimplant OR peri-implantitis OR “implant bone” OR “implant bone loss” OR “marginal bone loss”) AND (“artificial intelligence” OR “machine learning” OR “deep learning” OR “neural network” OR “convolutional neural network” OR CNN) AND (radiograph * OR “dental radiograph *” OR panoramic OR periapical OR orthopantomography * OR CBCT OR “cone beam”)) AND PUBYEAR > 2012 AND PUBYEAR < 2026	Publication year: 2013–2025

Search strategies used in PubMed and Scopus to identify studies on AI, radiographic imaging, and peri-implant bone conditions.

**Table 2:** Eligibility criteria.

Inclusion Criteria	Exclusion Criteria
Application of AI, machine learning, or deep learning to radiographic imaging of dental implants.	AI applications were not specifically related to dental implants.
Radiographic assessment of peri-implant bone conditions, including detection, classification, segmentation/measurement, or prediction of peri-implant bone loss or peri-implantitis.	Tasks not related to peri-implant bone assessment (e.g., implant detection, surgical planning, anatomical landmark identification, or generic dental image analysis).
Use of radiographic imaging modalities (periapical, panoramic, or CBCT).	Studies were not based on radiographic imaging data.
Original research studies evaluating or developing AI models.	Non-original publications (reviews, editorials, letters, conference abstracts, or opinion articles).
Human datasets involving dental implants.	Studies focusing on non-peri-implant conditions (e.g., periodontal disease, caries, orthodontics).
Articles published in English in peer-reviewed journals.	Insufficient methodological reporting (e.g., unclear dataset, model, or outcome evaluation).
Availability of a defined reference standard for peri-implant bone assessment.	
Reporting of quantitative performance metrics (e.g., accuracy, sensitivity, specificity, AUC, Dice coefficient, or measurement error).	
Focus on early or incipient peri-implant bone changes and/or predictive modeling of disease progression based on radiographic features.	

Predefined inclusion and exclusion criteria applied during study selection to identify eligible studies for the qualitative synthesis.

**Table 3:** Characteristics of the included studies.

Study (Author, Year)	Imaging Type	Task Type	AI Model	Dataset Size	Validation (Internal/External)	Performance Results	Clinical Relevance
Cha et al., 2021 [12]	Periapical radiographs	Detection + Measurement (with classification of severity)	ResNet-152 + Mask R-CNN (ResNet-FPN)	708	Internal	Bounding-box AP: 0.627 (upper), 0.657 (lower); keypoint AP: 0.761 (upper), 0.786 (lower) OKS comparable to dentist (ns)	Automated assessment of peri-implant bone loss for early detection and monitoring; adjunct tool
Chen et al., 2023 [16]	Periapical radiographs	Detection + Measurement (with classification of damage)	YOLOv2 + AlexNet CNN	456	Internal	YOLOv2 detection accuracy: 89.3%; AlexNet/CNN peri-implant damage classification accuracy: 90.45%	Automated evaluation of peri-implant tissue damage for postoperative monitoring; adjunct tool
Gao et al., 2025 [17]	Periapical radiographs	Detection/keypoint localization + measurement of marginal bone loss severity	YOLOv8-pose	208	Internal	Ppose: 0.999; mAP50–95 (pose): 0.994; most keypoint errors < 10 px; MBL severity accuracy: 0.906/0.844	Quantitative assessment of peri-implant marginal bone loss for monitoring and risk assessment; adjunct tool
Kibcak et al., 2025 [18]	Orthopantomographs	Detection/Classification (yes/no)	U-Net + AlexNet CNN	7696 OPGs; 3693 implant sites.	Internal	Segmentation: accuracy 0.999, DSC 0.986, IoU 0.974; classification: precision 0.777, recall 0.903, F1-score 0.835	Screening of peri-implantitis supporting diagnosis and treatment planning; adjunct tool
Lee et al., 2024 [15]	Periapical radiographs	Detection + Measurement (with severity classification)	YOLOv7	800	Internal	Accuracy 94.74%; precision 100%; recall 94.44%; specificity 100%; F1-score 97.10%; mAP 0.94	Assessment of bone loss severity enabling early diagnosis and standardized evaluation
Liu et al., 2022 [8]	Periapical radiographs	Detection of marginal bone loss	Faster R-CNN (Inception-ResNet v2)	1670	Internal	Sensitivity: 67–75%; specificity: 83–87%; mAP: 0.73; kappa: 0.55	Detection of peri-implant bone loss reducing diagnostic variability and clinician workload
Mao et al., 2025 [13]	Periapical radiographs	Detection + Measurement (with severity classification)	YOLOv8-S + image processing pipeline	780	Internal	YOLOv8-S detection: accuracy 98.7%, precision 98.1%, mAP50 99.2%; severity grading accuracy 95.8%; AUC 0.997–1.000; 36× faster than manual assessment.	Automated severity grading of peri-implant bone loss providing objective second-opinion assistance
Vera et al., 2023 [14]	Intraoral radiographs (periapical and bitewing)	Detection + measurement of peri-implant marginal bone remodeling	YOLOv3 + image processing pipeline	2336 (15% bitewing; 85% periapical)	Internal	mAP at IoU 0.5: 0.537–0.898; significant-point error: 2.63 ± 1.28 px (~0.17 ± 0.08 mm)	Quantitative assessment of bone remodeling for diagnosis and longitudinal monitoring
Zhang et al., 2023 [19]	Periapical and Orthopantomographs	Prediction of implant failure	ResNet-50 (hybrid model)	529 periapical + 551 OPGs, 248 implant sites	Internal	Hybrid model: accuracy 87.0%, precision 0.85, recall 0.88, F1-score 0.85; AUC 0.947–0.975	Prediction of implant failure risk enabling early intervention and closer monitoring
Lee et al., 2025 [20]	Orthopantomographs	Detection + Classification	YOLOv8 ensemble	1075 panoramic radiographs; 2250 implant sites	Internal	Overall accuracy 85.33%; precision 85.5%; recall 85.3%; F1-score 85.4%.	Detection and classification of peri-implant bone defects improving diagnostic consistency and decision-making

Summary of imaging modality, AI model, validation strategy, performance, and clinical relevance of the included studies. Abbreviations: AP, average precision; AUC, area under the curve; DSC, Dice similarity coefficient; IoU, intersection over union; mAP, mean average precision; MBL, marginal bone loss; OKS, object keypoint similarity; ns, not significant. Performance metrics are reported as presented in the original studies. Confidence intervals (CIs) were not consistently reported across the included studies and are therefore provided only when available in the original publications.

**Table 4:** CLAIM-based quality appraisal of the included studies.

Study	CLAIM Score	CLAIM Compliance (%)
Vera et al., 2023 [14]	33/40	82.5
Gao et al., 2025 [17]	31/40	77.5
Kibcak et al., 2025 [18]	30/40	75.0
Mao et al., 2025 [13]	30/40	75.0
Lee et al., 2025 [20]	30/41	73.2
Cha et al., 2021 [12]	29/40	72.5
Chen et al., 2023 [16]	28/40	70.0
Zhang et al., 2023 [19]	28/40	70.0
Lee et al., 2024 [15]	28/40	70.0
Liu et al., 2022 [8]	27/40	67.5

CLAIM, Checklist for Artificial Intelligence in Medical Imaging. Compliance scores were calculated as the percentage of reported CLAIM items among applicable items. Items considered not applicable were excluded from the denominator. Detailed item-level scores are reported in Supplementary Table S1.

## Data Availability

No new data were created or analyzed in this study. Data sharing is not applicable to this article.

## Supplementary Materials

The following supporting information can be downloaded at https://www.mdpi.com/article/10.3390/jcm15135210/s1. Table S1. Detailed evaluation of adherence to the 42 CLAIM reporting recommendations for each included study. Items were scored as reported (1), not reported (0), or not applicable (N/A). Overall CLAIM compliance was calculated as the proportion of reported items among applicable items. Figure S1. Reporting frequency of individual CLAIM items across the included studies. Green dots indicate high reporting frequency (≥80%), orange dots indicate moderate reporting frequency (30–79%), and red dots indicate low reporting frequency (<30%). The dashed horizontal line indicates the mean item-level CLAIM compliance across all assessed criteria. Items classified as not applicable were excluded from the denominator.
