# Supplementary material for: Artificial Intelligence for Radiographic Diagnosis of Peri-Implantitis: A Comprehensive Review on Detection, Measurement, and Risk Stratification
Source: J Clin Med. 2026 Jul 3;15(13):5210. doi: 10.3390/jcm15135210 (PMC13363319; doi:10.3390/jcm15135210)

Supplementary table S1. Detailed evaluation of adherence to the 42 CLAIM reporting recommendations for each included study. Items were scored as reported (1), not reported (0), or not applicable (N/A). Overall CLAIM compliance was calculated as the proportion of reported items among applicable items.

CLAIM item		Cha 2021	Chen 2023	Liu 2022	Zhang 2023	Vera 2023	Lee 2024	Gao 2025	Lee 2025	Kibcak 2025	Mao 2025
1	AI in title	1	1	1	1	1	1	1	1	1	1
2	Structured abstract	1	1	1	1	1	1	1	1	1	1
3	Clinical background	1	1	1	1	1	1	1	1	1	1
4	Objectives/hypothesis	1	1	1	1	1	1	1	1	1	1
5	Study design	1	1	1	1	1	1	1	1	1	1
6	Study goal	1	1	1	1	1	1	1	1	1	1
7	Data source	1	1	1	1	1	1	1	1	1	1
8	Eligibility criteria	1	1	1	1	1	1	1	1	1	1
9	Preprocessing	1	1	1	1	1	1	1	1	1	1
10	Data subset selection	1	1	1	1	1	1	1	1	1	1
11	Data definitions	1	1	1	1	1	1	1	1	1	1
12	De-identification	0	0	1	1	1	1	1	1	1	1
13	Missing data	N/A	N/A	N/A	N/A	N/A	N/A	N/A	N/A	N/A	N/A
14	Ground truth	1	1	1	1	1	1	1	1	1	1
15	Reference rationale	1	1	1	1	1	1	1	1	1	1
16	Annotator qualifications	1	1	1	1	1	1	1	1	1	1
17	Annotation tools	0	1	0	0	1	1	1	0	1	0
18	Inter/intra-rater agreement	1	0	1	0	0	0	0	1	1	0
19	Sample size	0	0	0	1	0	0	0	0	0	0
20	Data partitioning	1	1	1	1	1	1	1	1	1	1
21	Leakage prevention	0	0	0	0	1	0	1	1	0	1
22	Model architecture	1	1	1	1	1	1	1	1	1	1
23	Software/frameworks	1	1	1	1	1	0	1	1	1	1
24	Parameter initialization	1	1	0	1	1	1	1	1	1	0
25	Training details	1	1	1	1	1	1	1	1	1	1
26	Model selection	1	1	1	1	1	1	1	1	1	1
27	Ensemble methods	N/A	N/A	N/A	N/A	N/A	N/A	N/A	0	N/A	N/A
28	Performance metrics	1	1	1	1	1	1	1	1	1	1
29	Uncertainty estimates	1	0	0	0	1	0	0	0	0	1
30	Robustness analysis	0	0	0	0	1	0	0	0	0	1
31	Explainability	1	0	0	0	0	0	0	0	0	0
32	External validation	0	0	0	0	0	0	0	0	0	0
33	Flow diagram	1	1	1	1	1	1	1	1	1	0
34	Demographics by partition	0	0	0	0	0	0	0	0	0	0
35	Optimal model performance	1	1	1	1	1	1	1	1	1	1
36	Accuracy estimates (CI)	0	0	0	0	1	0	1	0	0	1
37	Failure analysis	0	1	0	0	1	1	1	1	1	1

38	Study limitations	1	1	1	1	1	1	1	1	1	1
39	Clinical implications	1	1	1	1	1	1	1	1	1	1
40	Registration	0	0	0	0	0	0	0	0	0	0
41	Protocol access	0	0	0	0	0	0	0	0	0	0
42	Funding/conflicts	1	1	1	1	1	1	1	1	1	1

Supplementary Figure S1. Reporting frequency of individual CLAIM items across the included studies. Green dots indicate high reporting frequency (≥80%), orange dots moderate reporting frequency (30–79%), and red dots low reporting frequency (<30%). The dashed horizontal line indicates the mean item-level CLAIM compliance across all assessed criteria. Items classified as not applicable were excluded from the denominator.

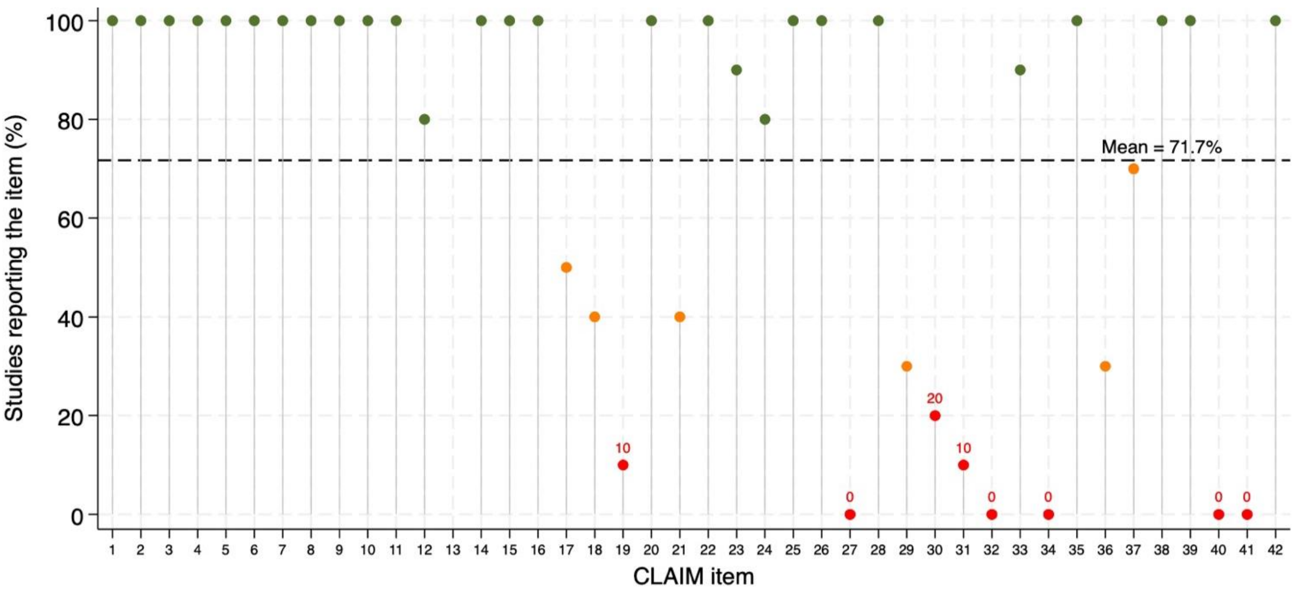

Supplement: Supplementary file 1 [file jcm-15-05210-s001.zip › jcm-4387240-supplementary.pdf]
